# Suppression of Shear Banding and Transition to Necking and Homogeneous Flow in Nanoglass Nanopillars

**DOI:** 10.1038/srep15611

**Published:** 2015-10-27

**Authors:** Sara Adibi, Paulo S. Branicio, Shailendra P. Joshi

**Affiliations:** 1 Institute of High Performance Computing, 1 Fusionopolis Way, #16-16 Connexis, Singapore 138632; 2 Department of Mechanical Engineering, National University of Singapore, 117576, Singapore

## Abstract

In order to improve the properties of metallic glasses (MG) a new type of MG structure, composed of nanoscale grains, referred to as nanoglass (NG), has been recently proposed. Here, we use large-scale molecular dynamics (MD) simulations of tensile loading to investigate the deformation and failure mechanisms of Cu_64_Zr_36_ NG nanopillars with large, experimentally accessible, 50 nm diameter. Our results reveal NG ductility and failure by necking below the average glassy grain size of 20 nm, in contrast to brittle failure by shear band propagation in MG nanopillars. Moreover, the results predict substantially larger ductility in NG nanopillars compared with previous predictions of MD simulations of bulk NG models with columnar grains. The results, in excellent agreement with experimental data, highlight the substantial enhancement of plasticity induced in experimentally relevant MG samples by the use of nanoglass architectures and point out to exciting novel applications of these materials.

Metallic glasses (MG) are the subject of an increasing number of studies due to their outstanding set of mechanical properties that includes high strength, high hardness, and high elastic energy storage capacity^1^,^2^,^3^,^4^,^5^,^6^,^7^,^8^,^9^,^10^. Their unique properties make them ideal candidates for a variety of structural applications^11^,^12^,^13^. A major weakness of MGs is their lack of macroscopic ductility arising from their propensity to shear localization^4^. Current research on MGs is focused on finding avenues to mitigate localized plasticity by spreading out plastic deformation through the material volume raising the overall ductility and toughness of the samples.

Different methods have been reported to augment the tensile ductility of MGs, such as pre-deformation of samples and insertion of nanocrystalline inclusions^14^,^15^. The use of such methods results in a pattern of multiple SBs distributed in the MG sample, which carry on plasticity and increase the overall ductility of the sample during the deformation. Recently, an exciting new approach has been proposed and may enhance significantly the ductility of MGs^16^. In this approach, not involving changes of composition or pre-processing, fine MG powder is consolidated by cold compression, generating what is called a nanoglass (NG) architecture. The inherent glass-glass interfaces (GGI) in a NG were shown to have structural similarities to SBs^17^,^18^,^19^,^20^,^21^. They exhibit higher free volume (indicated by the atomic Voronoi volume) and lower short range atomic order when compared to the glassy grains, and can act as preferred channels for plasticity^22^,^23^. Molecular-dynamics (MD) simulations of the deformation of NGs have shown promising results^22^,^23^,^24^,^25^,^26^,^27^,^28^, indicating that in fact the plasticity of NGs is substantially improved compared to their MG counterparts. However, these early works have focused on bulk two-dimensional (2D) like geometries, most of them making use of fully periodic boundary conditions (PBCs), and may be influenced by constraint effects^22^,^23^,^26^,^27^. It remains to be validated if the improved plasticity reported in these simulations is also present in more realistic three-dimensional (3D) systems of experimental relevance. Such validations can be provided by large scale MD simulations of models such as nanopillars. For instance, MG nanopillars have been experimentally widely used to investigate the intrinsic failure of MGs^5^,^6^,^8^,^26^,^29^,^30^,^31^,^32^,^33^. Very recently, Wang *et al.*^33^ also reported the deformation and the failure of NG nanopillars.

In this work, we report on large-scale MD simulations of tensile loading of Cu_64_Zr_36_ MG and NG cylindrical nanopillars with 50 nm diameter and aspect ratio 2.5 (nearly 16 million atoms). We evaluate the effect of glassy grain size (*d* = 3 to 20 nm) on the ductility and failure of NG nanopillars. We find that NG nanopillars possess significant macroscopic ductility and exhibit necking induced failure at all grain sizes, in striking contrast with the observed brittle failure in the MG nanopillars. In addition, the results reveal monotonic increase of homogeneous plasticity with decreasing grain size, which tends to near superplastic flow at *d* < 5 nm.

Nanopillars with diameters of 100 nm or smaller can be readily fabricated with state of the art focused ion beam or electroplating^29^,^31^,^34^,^35^. Large scale MD simulations can access systems with millions to billions of atoms^36^,^37^,^38^,^39^, which enable modelling nano-scale geometries that match similar experimental samples^40^. Here, we simulate the tensile loading of Cu_64_Zr_36_ MG (used as reference) and NG cylindrical nanopillars of diameter (*D*) 50 nm and length (*L*) 125 nm. To investigate the role of grain size in NGs, we consider the range 3 ≤ *d* ≤ 20 nm (Fig. 1). In the figure, each grain is shown with different color in order to highlight the nanostructured nature of the NGs. In our MD simulations we adopt a time step Δt ≤ 5 fs in the integration of the equations of motion. The atomic interactions are calculated using an embedded atom model potential fitted to CuZr properties^41^.

Bulk NG samples with desired average grain sizes used to produce the NG nanopillars are initially generated using a procedure employed previously^17^,^27^. NG samples are constructed based on the Poisson-Voronoi tessellation method^42^,^43^,^44^. In this procedure, NG grains are generated from a reference MG structure, which is produced following a method detailed elsewhere^17^,^27^. Both the reference MG system and the generated NG model have identical dimensions. Grains in the NG sample are filled up with a corresponding volume of material taken from the MG reference system. To produce NG interfaces the grains are filled one by one and all atoms in the original periodic MG system are translated by applying a random shift to the atomic positions after each grain is filled. To avoid overlapping of atoms at interfaces grains are filled up to 1 Å from the mathematically defined interface planes. In addition, after the NG sample is produced atoms at interfaces are removed to ensure that no pair of atoms is closer than 2.2 Å. This distance threshold is based on the average nearest neighbour distances found in bulk CuZr MG, which are 2.7 Å, 3 Å, and 3.1 Å for Cu-Cu, Cu-Zr, and Zr-Zr pairs, respectively. Following this, the NG models are consolidated and relaxed by cold compression. A high hydrostatic pressure of 3 GPa is applied to the system at 50 K for 0.04 ns in order to relax the atomic structure of the interfaces and minimize the initial porosity. The cold compression is followed by additional relaxation of the system at zero pressure and 50 K for 0.04 ns. The NG nanopillars are finally produced by carving its volume from the relaxed bulk NG system. Free surfaces of the nanopillars are relaxed using Langevin Dynamics for 0.02 ns. Residual stresses in the nanopillars axis (*z* direction) are relaxed to σ_zz_ = 0 using the NPT ensemble for 0.02 ns. Samples are simulated under uniaxial tensile loading. PBCs are applied along the cylinders axis (*z* direction), while free surfaces are used in the lateral, *x* and *y* directions. The traction-free lateral surfaces ensure that the specimens experience a uniaxial stress state. We use a tensile strain rate, 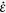 = 4 × 10^8^ s^−1^, along the *z* direction. During loading, the system temperature is maintained at 50 K. Engineering stress is calculated computing the average atomic Virial stress in the system^45^. The generation and evolution of local inelastic deformation is mined using the atomic local von Mises shear strain ε_M_^46^. MD simulations are performed using LAMMPS^47^ and the results are visualized using OVITO^48^.

Figure 2a shows the engineering stress-strain curves calculated from the simulations of deformation of each NG nanopillar. The reference stress-strain curve for the MG nanopillar shows a typical drastic stress drop at ε ≈ 0.07, which is an indication of brittle failure by initiation and propagation of a single SB. It should be noted that the finite stress drop at the yield point is an effect of the use of fixed strain rate deformation and a low *L*/*D* = 2.5. For reasonably high *L*/*D* (>13) one should expect a rapid drop to zero stress signifying realistic *brittle* failure. In contrast, NG nanopillars exhibit smoother stress drops beyond yield (peak stress), indicative of stable macroscopic plasticity. It can be seen that the decrease in the glassy grain size induces both, a lower yield (peak) stress and a decreasing rate of stress drop post yield. This suggests a transition in the failure mechanism from MG to NG nanopillars. Figure 2b quantifies the inverse dependence of the peak stress on NG grain size. Interestingly, this *yield softening* depends nonlinearly with grain size with faster drop for *d* ≤ 10 nm. It is useful to note however, that all NG nanopillars preserve more that 40 % of the initial predicted MG intrinsic strength (NGs’ yield strength >1.5 GPa) while suggesting significant induction of plasticity.

Interestingly, for the extreme case of the NG with *d* = 3 nm, the stress beyond yield increases mildly before starts to drop at a nominal strain ε = 0.3, which implies strain hardening. To better quantify the strain hardening we calculated the true stress-true strain curve, shown in Fig. 2c. We can see strain hardening from the yield point till ε = 0.25. This NG behavior was not previously predicted by bulk NG simulations^17^,^27^ and highlight the importance of simulating realistic system geometries.

In order to understand the change in the deformation mechanism experienced by NG nanopillars with different *d*, we examine the atomic deformation processes by analyzing the distribution of atomic local von Mises strain^46^, ε_M_ which is calculated with respect to the relaxed configurations prior to loading. Figure 3 shows a sequence of snapshots illustrating the atomic deformation processes for the MG and NG nanopillars. In MG nanopillar (Fig. 3a), few regions with high ε_M_ emerge, indicating strain localization and the generation of embryonic SBs. With progressive deformation, one of the SBs propagates rapidly thereby causing catastrophic brittle failure. In contrast, for the NG nanopillars (Fig. 3b–f) regions of high strain follow the GGI motif and they remain so with increasing deformation. In other words, NG GGIs act as preferred channels that carry plastic deformation and distribute it throughout the nanopillar volume. Interestingly, NG nanopillars exhibit necking instability at all grain sizes, in contrast to the shear localization observed in MGs. A direct consequence of necking is the smoother decay of stress from the yield point, as illustrated in Fig 2a. It can be seen that by decreasing *d* from 20 to 3 nm, the finer GGI network results in a more distributed plastic deformation. For the extreme case of NG nanopillar with *d* = 3 nm the very dense GGI network generates homogeneous flow until ε ~ 0.33, resulting in a near homogeneous deformation.

In order to quantify the degree of strain localization in MG and NG nanopillars, we calculate the deformation participation ratio (DPR)^49^, which gives the fraction of atoms that undergoes a local atomic shear deformation higher than the nominal strain. For a specimen undergoing homogeneous plastic deformation DPR 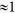. Figure 4a shows the DPR for the NG nanopillars at ε = 0.11. It can be seen that the DPR increases with decreasing *d*. For MG and for NG with *d* = 20 nm the DPR ~ 0.35 implying localized plastic deformation. However, one should note that the observed localization of plastic deformations in MG and NG nanopillars with *d* = 20 nm is fundamentally different (Fig. 3). As can be seen in Fig. 3a localization in the former occurs within a single shear band that crosses the whole system. On the other hand, for the latter localization occurs within the network of GGIs. To further quantify the fraction of the atoms involved in the plastic deformation during the strain loading we calculate the fraction of atoms with relatively high local atomic shear strain. Figure 4b shows the fraction of atoms with ε_M_ > 0.2 as a function of strain. The threshold value of 0.2 was chosen based on the distribution of the von Mises strain for all the atoms in the system (see Supplementary Fig. 1). Compared to the MG case, NGs have a higher fraction of atoms that undergo plastic shear strain or structural changes due to the presence of GGIs. In addition, with decreasing grain size, the fraction of atoms with high shear strain increases. This is expected, since the decreasing in glassy grain size results in increasing GGI fraction with concomitant increase in the volume that is able to carry higher shear strain. During deformation, the fraction of atoms undergoing large shear deformation reaches nearly 90% for NG with *d* = 3 nm, implying that a substantial fraction of the material volume experiences structural changes that promote near homogeneous plastic flow.

Since the structure of the NG GGIs is distinct from the structure of the grains (bulk), and GGIs play an active role in the initiation of SBs, it is instructive to calculate the fraction of interfacial material in a NG as a function of grain size. To that end, we assume a GGI thickness of 1.38 nm, as estimated in our recent work^27^. Based on this, we calculate the fraction of material at bulk, GGIs, grain boundaries (flat GGIs), and triple and higher junctions, as a function of grain size. Figure 5 shows that the GGI fraction is significantly high for *d* = 3 nm (~91%), while for *d* = 20 nm, it is only ~21%. While the GGI fraction increases as the grain size decreases, the grain boundary fraction does not have the same trend. The data show that grain boundary fraction increase by decreasing grain size until *d* ~ 6 nm and then starts to decline. This trend shows the importance of triple and higher junctions at finer grain sizes. It can be seen that the crossover between the bulk and GGI fractions occurs at *d* ~ 8 nm, while the crossover of bulk, grain boundaries, and triple and higher junctions’ fractions occurs at *d* ~ 5.5 nm. In a recent work, we show that the statistics of Voronoi polyhedra of the Cu_64_Zr_36_ GGIs are similar to that present in SBs^27^. In particular, it reveals significantly less fraction of Cu-centered full icosahedra in the GGIs, which are usually associated with the strong-but-brittle behavior of Cu-rich CuZr MG^4^. Therefore, the crossover of the bulk and GGI fractions at *d* ~ 8 nm indicates that NG nanopillars with grain size at this range or below should experience large ductility. This clearly is the case as shown by the trends observed in Fig. 3, which indicate that for *d* < 10 nm a significant, near homogeneous plastic deformation, occurs before failure by necking. In nanocrystalline materials, homogeneous deformation at room temperature may occur by activation of several mechanisms which include grain boundary sliding, grain rotation and grain boundary diffusion^50^. Although, we have not evaluated the presence of similar atomistic mechanisms in the context of NGs, the results shown in Fig. 3e,f motivate a separate investigation on this topic.

It is worth noting that an unexpected characteristic of the 3D NG structure of the nanopillars is that it is ductile at all grain sizes and the observed ductility is enhanced for decreasing grain sizes. As the grain size is reduced, we clearly observe a significant increase in the extent of homogeneous deformation in NG nanopillars. As discussed previously, the interfacial regions play a very important role in the induced NG plasticity. The higher plasticity shown in the NG nanopillars at a smaller grain size is directly related to the higher fraction of interfacial material. That can be verified comparing the fraction of interfacial materials between NG nanopillars with different grain sizes, Fig. 5. Therefore, the results indicate that the fraction of GGI material is a critical parameter affecting the ductility in NG architectures. It should be highlighted here that the nanoglass small diameter *per se* has no effect on the observed ductility of the NG nanopillars. There are several reports of brittle-to-ductile in nanopillars due to reduction in diameter^51^,^52^,^53^. In particular, our recent work^54^ demonstrates that nanoscale specimens with no structural heterogeneities, such as surface imperfections, always fail through catastrophic shear banding, even at diameters as small as 10 nm^55^. Further, as can be observed in Fig. 3 a MG nanopillar of similar dimensions as the nanoglasses fails via brittle mode by propagation of a single shear band. From these observations, the substantial ductility observed in the NG nanopillars is attributed solely to its inbuilt nanoglass design.

It is instructive to compare the predictions presented here for NG nanopillar architectures to the predications for bulk NG samples reported previously^23^,^26^,^27^. As noted in the introduction, prior MD simulations on bulk NGs employed columnar glassy grains (2D like architectures). Together with constraints from PBCs mimicking bulk systems, such architectures may strongly suppress necking. Therefore, those results may not correspond directly to realistic scenarios involving deformation of pillars. To demonstrate this, we simulate bulk 2D like NG architectures, as in previous works, over the same glassy grain size range as those used in the NG nanopillars, at 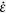 = 4 × 10^8^ s^−1^. For brevity, these results are consolidated in Supplementary Information. The salient features that differ those architectures from NG nanopillars are: (i) transition from SB to homogeneous flow with decreasing grain size, and (ii) absence of necking at all grain sizes (Fig. S3).

The foregoing results allude to the prospect of designing NG architectures exhibiting tensile ductility with relatively coarse grain sizes (in the range of tens of nm or more). Even though necking is not constrained, as in the case of 2D like architectures, it is surprising to observe its development in all samples. This is a key result of this work that indicates that the induced ductility by NG design is much more intense than previously conceived. In previous investigations of bulk NG samples with columnar-grains the induced ductility was rooted at the delocalization of plastic deformation along GGIs. Eventually the bulk NG samples would fail by shear banding as a typical MG system, unless the glassy grain size was in the extreme small range *d* ≤ 5 nm. In the nanopillar case the induced ductility still follows the delocalization pattern predicted for bulk samples. However, shear banding is suppressed at all glassy grain size investigated leading to failure by necking. We note that the tensile loading conditions in the nanopillars and bulk samples considered are identical. Furthermore, the average grain size range in both, bulk samples and NG nanopillars is the same. By deductive argument, the parameter that correlates to this SB suppression in the latter is the nature of GGI connectivity. In the bulk samples, GGI connectivity is restrictive (2D) owing to the columnar grain structure (Fig. S3b). On the other hand, NG nanopillars possess GGI connectivity that is 3D in nature. Comparing the GGI fraction in NG nanopillars (Fig. 5) with the one for columnar architectures reported recently^27^, we observe the former have nearly 19%, 63% and 100% larger GGI fraction compared to the latter at grain sizes 15, 10, and 5 nm, respectively. In particular, due to the 3D Poisson Voronoi tessellation NG nanopillar structures have a higher volume fraction of GGIs and triple junctions than the bulk columnar grain structure at the same grain size. In addition, NG nanopillars possess tetra- and higher junctions, which are absent in the bulk samples. These differences may explain the nature of plasticity in the NG nanopillars at these grain sizes. The plastic deformation, which preferentially occurs along the GGIs, is not spatially restricted to 2D in nanopillars, which should result in a triaxial stress state that favors necking. This is plausible because, regions of high plastic strain (e.g. SBs and GGIs) experience high hydrostatic stress (under tensile loading), which may cause enhanced free volume and void evolution^56^,^57^.

Recently, two experimental works on NG were reported. Franke *et al.*^58^ investigated the thermal and plastic properties of FeSc NG films. They employed nanoindentation tests to probe the incipient plasticity and the influence of NG interfaces. Their results indicate that NG interfaces are very stable at room temperature and significantly influence the mechanical properties by suppressing shear localization (formation and propagation of shear bands). These conclusions are in excellent agreement with our current and previous work on NG^26^,^27^. In a more recent work, Wang *et al.*^33^ probed the mechanical behavior of Sc_75_Fe_25_ NG nanopillars using *in situ* tensile testing inside a transmission electron microscope. This work serves as a key experimental counterpart to our work and the results reported here. Wang *et al.* used comparable NG sample geometries (nanopillars), dimensions (400 nm diameter), and grain sizes (10 nm) as those used in our work. Their results are in good agreement with our predictions. Their main results indicate that NG nanopillars deformation resembles that of a ductile material: (i) extensive macroscopic plastic deformation under uniaxial tension ~15% and ultimate failure by necking, which corroborates very well with our predictions (cf. Figs 2 and 3); (ii) ~28% lower yield stress for NG specimens than the MG counterparts, which is comparable to our ~33% yield softening between MG and NG with *d* = 10 nm. In addition to the outstanding agreement of results, the work of Wang *et al.* is particularly important to our NG modeling since it sheds light into the microstructure of the samples, which are strikingly similar to the ones produced in our work. For instance, the soft interfaces reported by Wang *et al.* are ~1 nm thick, which agrees well with our predicted interface thickness of 1.34 nm. The reported interfaces also undergo a thickening process during the plastic deformation indicating they are active soft channels for plasticity in dramatic similarity with our results. This set of experimental data gives confidence that the NG models simulated are realistic and describe accurately the properties of NG.

As a final note, we should highlight that our results, in good agreement with experimental data, point out that NG design enhances significantly the ductility of MG and open the field to wide spread investigation into designing and synthesizing nanoglass microstructures that are tuned to applications. However, the first investigations of nanoglasses used inert-gas condensation or magnetron sputtering as a way to produce nanometer-sized grains. Such methods are convenient to generate NG thin films that could be applied to nanodevices, such as NEMS and MEMS. Nonetheless, such samples preclude any bulk application of NG. An alternative method for producing bulk quantities of NG is therefore needed. The similarity of properties of the glass-glass interfaces to that of shear bands^27^ offers a natural alternative path to produce nanoglasses. By applying severe plastic deformation to a bulk MG sample, e.g. ball milling, a nanoglass like microstructure can be produced by introducing a high density of shear bands. Such approaches may provide viable paths to produce bulk quantities of NG that would have wide spread use as an alternative to traditional bulk MG. Nevertheless, preliminary attempts of using such process showed contrasting results indicating that further work is required in this direction^21^.

In summary, our MD simulation results of tensile loading deformation of Cu_64_Zr_36_ NG nanopillars reveal that NG microstructures exhibit macroscopic plasticity and ductile failure by necking over a wide range of glassy grain sizes. In addition, the results show an increasing delocalization of plastic deformation on grain size reduction leading to near superplastic flow at *d* < 5 nm. These predictions for experimentally realizable NG nanopillar structures contrast with those made previously for NG bulk samples. In particular, NG nanopillars show enhanced ductility at all grain sizes due to the characteristic of the 3D grain structure, which enhances the fraction of interfacial material and suppress shear band propagation. All the predictions are in excellent agreement with recent experiments on NG nanopillars. These exciting simulation predictions validated by experimental data suggest that MGs with exceptional plasticity may be conveniently generated by using NG design. That points to novel and wide spread structural applications of MG in the near future.

## Additional Information

**How to cite this article**: Adibi, S. *et al.* Suppression of Shear Banding and Transition to Necking and Homogeneous Flow in Nanoglass Nanopillars. *Sci. Rep.* **5**, 15611; doi: 10.1038/srep15611 (2015).

## Supplementary Material

Supplementary Information

Supplementary Movie S1

Supplementary Movie S2

Supplementary Movie S3

Supplementary Movie S4

Supplementary Movie S5

Supplementary Movie S6

Supplementary Movie S7

Supplementary Movie S8

Supplementary Movie S9

Supplementary Movie S10

Supplementary Movie S11

Supplementary Movie S12

## Figures and Tables

**Figure 1:**
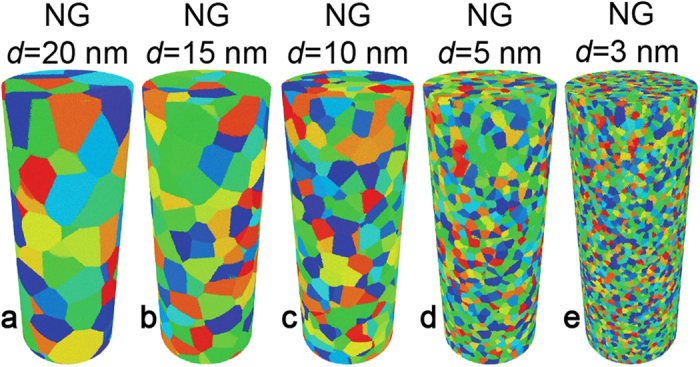
Nanoglass (NG) nanopillars used in the simulations. (**a**)-(**e**) illustrations of nanopillars with 50 nm diameter and average grain sizes *d* = 20, 15, 10, 5, and 3 nm. Grains are shown in different colors to highlight the nanostructure.

**Figure 2:**
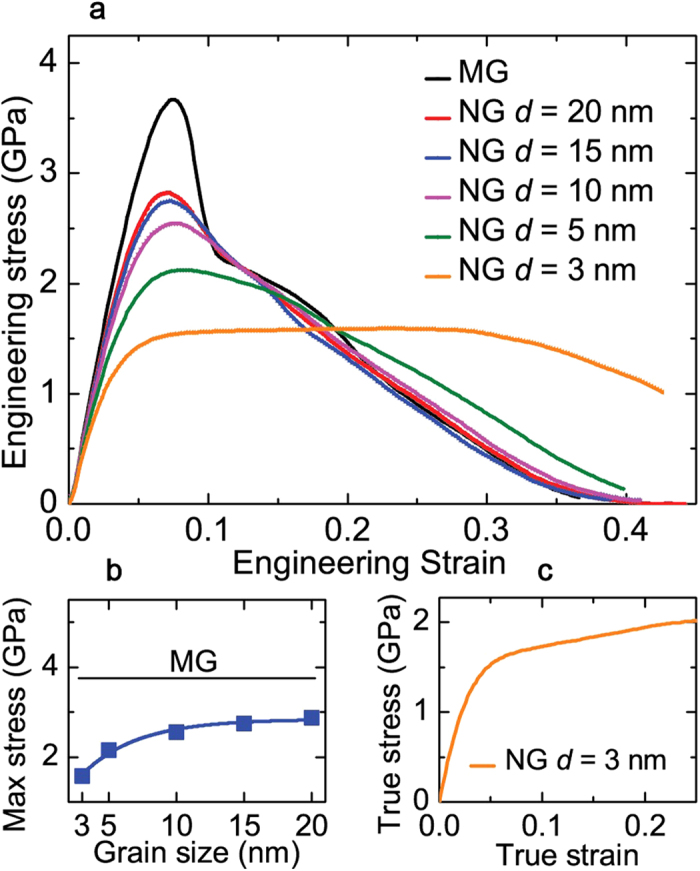
Grain size effect on tensile stress-strain curves of Cu64Zr36 metallic glass (MG) and NG nanopillars. (**a**) Engineering stress-strain curves for the MG nanopillar and the NG nanopillars with *d* = 20, 15, 10, 5, and 3 nm. (**b**) Maximum stress vs d from the curves shown in (**a**). The value of the maximum stress for the MG is also shown for reference. (**c**) True stress-strain curve for the NG nanopillar with *d* = 3 nm.

**Figure 3:**
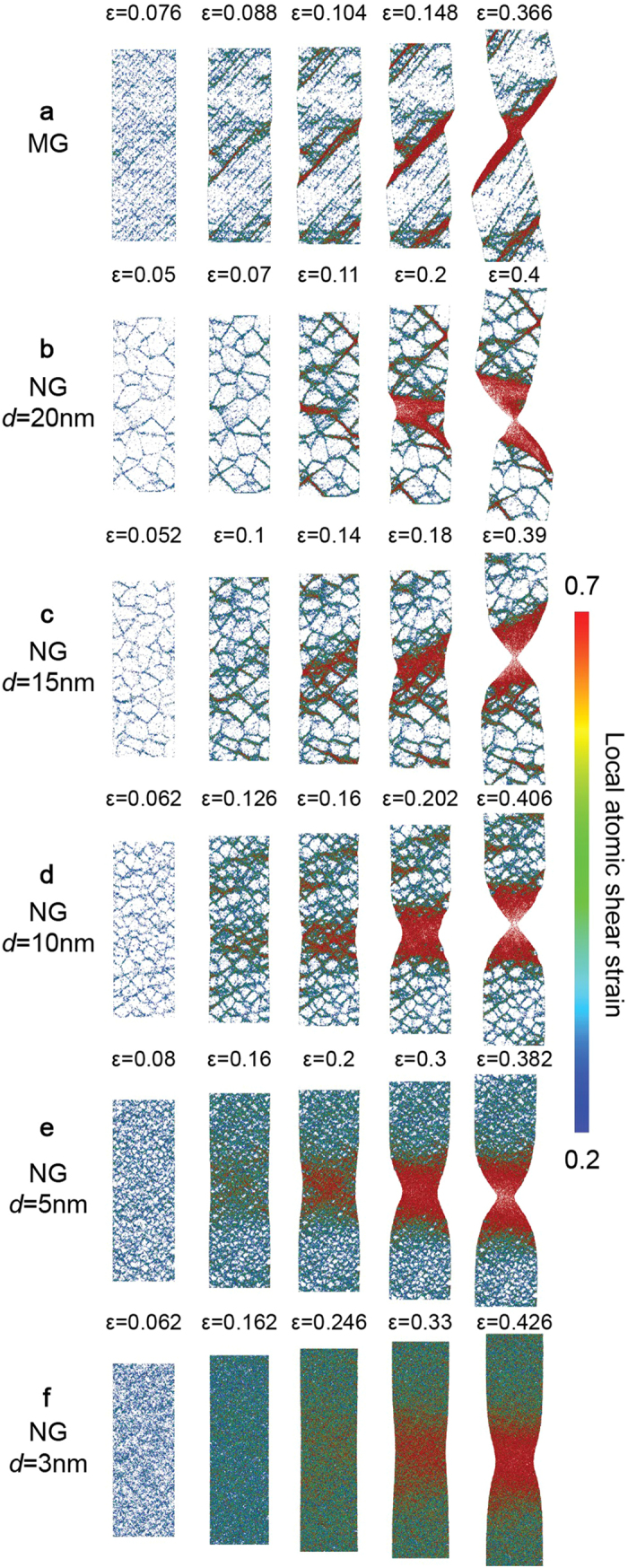
Illustrations of the deformation and failure of MG and NG nanopillars. (**a**)–(**f**) Sequence of snapshots capturing the atomic deformation processes for MG nanopillar and NG nanopillars with *d* = 20, 15, 10, 5, and 3 nm, respectively. The color indicates the local atomic shear strain. For clarity, only atoms with local atomic shear strain higher than 0.2 are shown. Illustrations are produced from visualizations of a 1 nm thick slice from the middle part of the nanopillars.

**Figure 4:**
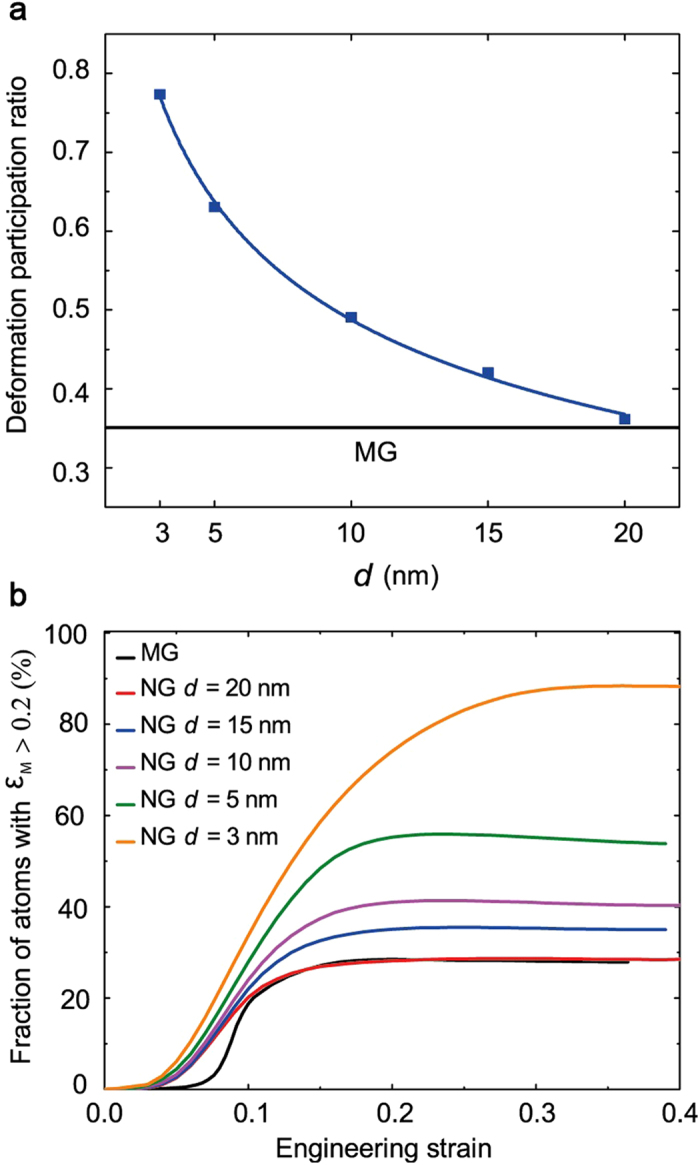
Analysis of atomic deformation engagement in MG and NG nanopillars of different grain sizes. (**a**) Deformation Participation Ratio (DPR) at macroscopic strain ε = 0.11. (**b**) Fraction of atoms with ε_M_ ≥ 0.2 during deformation.

**Figure 5:**
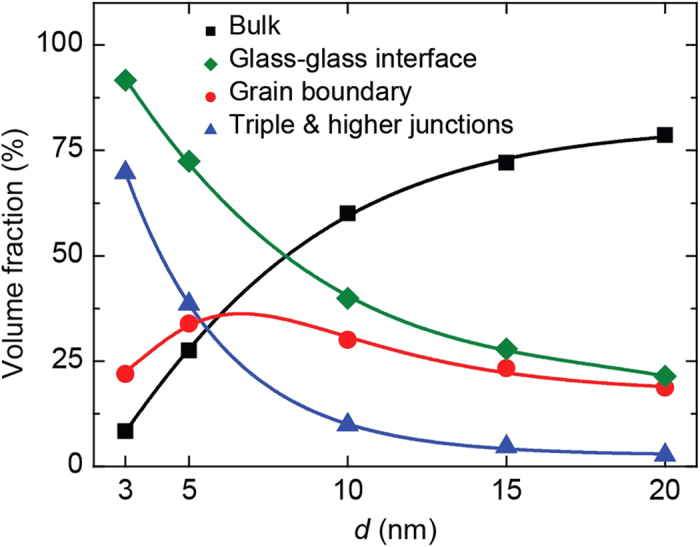
Fraction of atoms in MG grains (bulk) and NG interfacial regions as a function of grain size. Atoms in glass-glass interfaces include those in grain boundaries, and triple and higher junctions.
