# Supplementary material for: Suppression of Shear Banding and Transition to Necking and Homogeneous Flow in Nanoglass Nanopillars
Source: Sci Rep. 2015 Oct 27;5:15611. doi: 10.1038/srep15611 (PMC4621512; doi:10.1038/srep15611)
Supplement: Supplementary Information [file srep15611-s1.pdf]

SUPPLEMENTARY INFORMATION

Suppression of Shear Banding and Transition to Necking and Homogeneous Flow in Nanoglass Nanopillars

Sara Adibi,[1,2] *Paulo S. Branicio*[1*], *Shailendra P. Joshi*[2]

[1]Institute of High Performance Computing, 1 Fusionopolis Way, #16-16 Connexis, Singapore 138632

[2]Department of Mechanical Engineering, National University of Singapore, 117576, Singapore

KEYWORDS: ductility, nanoglass, metallic glass, grain size, molecular dynamics simulations

[*]E-mail: branicio@ihpc.a-star.edu.sg. Phone: +65-6419-1237

Local atomic shear strain distribution

In order to further improve upon the visual cues of deformation mechanisms in MG and NGs, we quantify the fraction of atoms at different local atomic shear strains. Supplementary figs 1a and 1b show the normalized distribution of atoms according to their local atomic shear strain (ε_M) at engineering strains of 0.11 and 0.35, respectively. In the calculation, we use a histogram of bin size 0.01 and the total number of atoms normalize the values in each system. For each grain size, the corresponding histogram exhibits a characteristic unimodal distribution with a peak at atomic shear strain less than or near the applied macroscopic strain. The full width at half maximum of the distributions increases for decreasing grain size. That indicates a gradual increase in the fraction of atoms undergoing plastic deformation as the grain size is reduced. One may note that for NG with d = 3 nm the peak in the fraction of atoms is at a value near the engineering strain, indicating a near-homogeneous shear strain distribution in the sample. Based on the peaks shown in Supplementary figs 1a and 1b we chose a threshold value of $\varepsilon_M = 0.2$ to select the atoms participating in the plastic deformation.

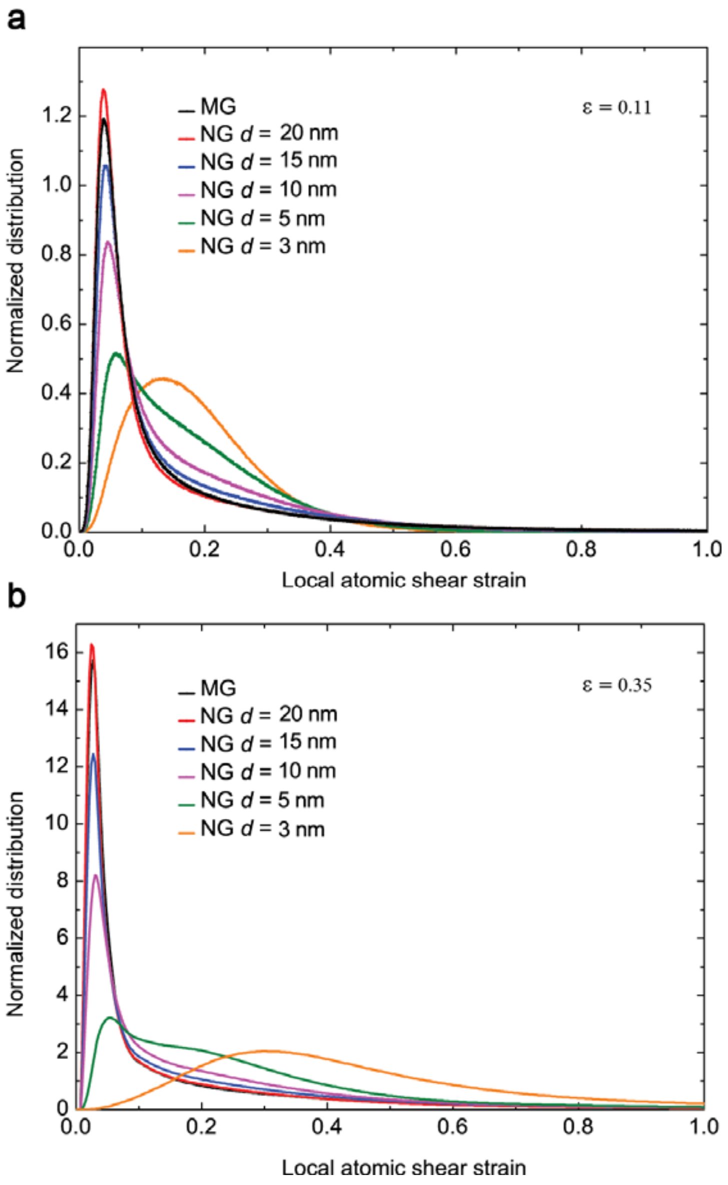

Supplementary Figure 1. Normalized distribution of local atomic shear strain at (a) $\varepsilon = 0.11$, and (b) $\varepsilon = 0.35$. Values are calculated using a histogram of bin size 0.01 and normalized by the total number of atoms. The threshold value, $\varepsilon_M > 0.2$, is chosen to select the atoms with high local atomic shear strain.

Comparison of NG designs: bulk with columnar-grain structure vs nanopillar with 3D grain structure

In this section, we present the deformation failure characteristic in bulk NG bulk samples with columnar grain structure. This kind of structure was previously reported in the literature.[1, 2 3] Therefore, it is important to compare the predictions of this work for NG nanopillars to those bulk NG reference studies. To have a fair comparison between the deformation of NG bulk samples and those of NG nanopillars, we investigated the deformation of NG bulk samples at strain rate of 4×10^{8} s^{-1}, the same one used in this study.

Supplementary Figure 2 shows the stress-strain response of the NG bulk samples. It can be seen that the NG bulk samples with $d \geq 10$ nm show the typically brittle stress drop after yield point. However, the NG with d = 5 nm shows a very gradual decay in the stress-strain curve. NG with d = 3 nm shows a flat stress-strain curve after the yield point which is an indication of homogeneous deformation in the sample.

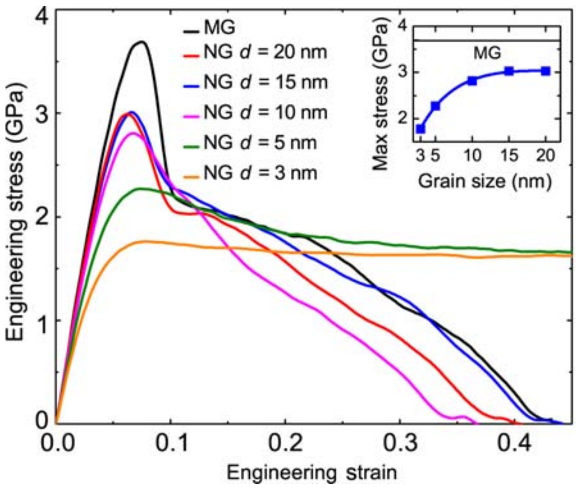

Supplementary Figure 2 | Engineering stress-strain curves for MG and NG bulk samples with *d* = 20, 15, 10, 5, and 3 nm. The inset shows the maximum stress as a fuction of *d*.

In order to gain atomistic insights over the deformation mechanisms in NG bulk samples, we show in Supplementary Figure 3 snapshots of the atomic structures of the MG and NG bulk samples. Atoms are colored according to their local von Mises shear strain. At strain ~ 0.08, immediately after the major stress drop (see Supplementary Figure 2), a path of high strain is generated across the width of the NG bulk samples with $d \geq 10$ nm. This localization path is formed by a combination of high strain regions located at GGIs. The strain along this path intensifies with progressive straining, as shown by the shift of the colors in Supplementary Figure 3 from light blue to dark red. The intensification of strain also appears as a thickening of the GGI strain path. Following the strain loading, a single dominant shear band eventually develops in the systems. However, for NG bulk samples with $d \leq 5$ nm, due to the high density of GGIs, a highly delocalized distribution of plastic deformation is observed. In contrast with the case of $d \geq 10$ nm, here the deformation remains delocalized leading to homogeneous deformation of the NG samples. From previous analysis of atomic fractions at GGIs, it is clear that a smaller glassy grain size leads to a larger volume fraction of GGIs,[1] facilitating the delocalization of plastic strain in the sample. Therefore, the simulation on NG bulk samples (see Supplementary Figure 3) shows a transition from localized SB formation to homogeneous deformation at the threshold value $d = 5$ nm.

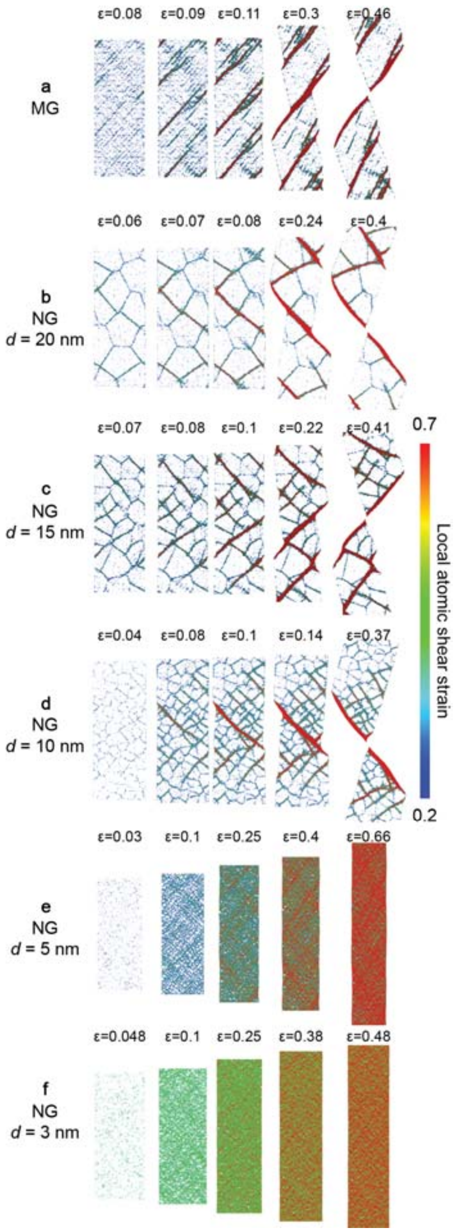

Supplementary Figure 3 | Illustrations of the deformation and failure of MG and NG bulk samples. (**a**)–(**f**) Sequence of snapshots capturing the atomic deformation processes for MG and NG bulk samples with d = 20, 15, 10, 5, and 3 nm, respectively. The color indicates the local atomic shear strain. For clarity, only atoms with local atomic shear strain higher than 0.2 are shown.

Supplementary movies

Deformation profiles os samples under tensile loading visualized by van Mises local shear strain:

Movie S1. MG nanopillar.
Movie S2. NG nanopillar with d = 20 nm.
Movie S3. NG nanopillar with d = 15 nm.
Movie S4. NG nanopillar with d = 10 nm.
Movie S5. NG nanopillar with d = 5 nm.
Movie S6. NG nanopillar with d = 3 nm.
Movie S7. MG bulk.
Movie S8. NG bulk d = 20 nm.
Movie S9. NG bulk d = 15 nm.
Movie S10. NG bulk d = 10 nm.
Movie S11. NG bulk d = 5 nm.
Movie S12. NG bulk d = 3 nm.

References

1. Adibi S, Branicio PS, Zhang Y-W, Joshi SP. Composition and grain size effects on the structural and mechanical properties of CuZr nanoglasses. *J Appl Phys* **116**, 043522 (2014).

2. Adibi S, et al. A transition from localized shear banding to homogeneous superplastic flow in nanoglass. *Appl Phys Lett* **103**, 211905 (2013).

3. Şopu D, Ritter Y, Gleiter H, Albe K. Deformation behavior of bulk and nanostructured metallic glasses studied via molecular dynamics simulations. *Phys Rev B* **83**, 100202 (2011).
